# Complete Genome Sequence of *Bacteroidales* Strain CF from a Chloroform-Dechlorinating Enrichment Culture

**DOI:** 10.1128/genomeA.01066-13

**Published:** 2013-12-19

**Authors:** Shuiquan Tang, Elizabeth A. Edwards

**Affiliations:** Department of Chemical Engineering and Applied Chemistry, University of Toronto, Toronto, Ontario, Canadaa; Department of Cell and Systems Biology, University of Toronto, Toronto, Ontario, Canadab

## Abstract

*Bacteroidales* strain CF is the most abundant nondechlorinating organism in a *Dehalobacter*-containing enrichment culture that consistently reductively dechlorinates >50 mg/liter chloroform or 1,1,1-trichloroethane (methyl chloroform). We assembled and closed the complete genome sequence of this organism from the metagenomic sequencing data for enrichment cultures. This organism is predicted to ferment l-lactate and ethanol.

## GENOME ANNOUNCEMENT

ACT-3 is an enrichment culture used for bioaugmentation to detoxify groundwater contaminated with 1,1,1-trichloroethane (1,1,1-TCA), 1,1-dichloroethane (1,1-DCA), or chloroform (CF) (1). It is grown in prereduced defined mineral medium amended with ~50 mg/liter 1,1,1-TCA as an electron acceptor and a mixture of methanol, ethanol, and lactate as electron donors. The ACT-3 culture is dominated by two *Dehalobacter* strains that make up ~70% of the community. In this culture, 1,1,1-TCA is reductively dechlorinated via 1,1-DCA to monochloroethane and CF is reductively dechlorinated to dichloromethane (2). A subculture of the ACT-3 culture is maintained identically except that 1,1,1-TCA is replaced by CF. This subculture is dominated (~80%) by only one of the two *Dehalobacter* strains from the parent culture (3). The parent and CF subcultures also share a nondechlorinating organism that is the next most abundant (8 to 9%) in both cultures (3). We have named this organism *Bacteroidales* strain CF based on 16S rRNA phylogeny. This organism is of interest because it ferments in the presence of concentrations of CF or 1,1,1-TCA that usually inhibit microbial processes, including methanogenesis (4, 5) and reductive dechlorination (6, 7).

The complete genome sequence of *Bacteroidales* strain CF was assembled from the metagenomes of ACT-3 and the CF subculture. ACT-3 was sequenced using 454 pyrosequencing, including paired-end sequencing of an 8-kb insert library, and the sequence was assembled with Newbler version 2.5 (8). The CF subculture was sequenced using paired-end Illumina technology, and the sequence was assembled with AbySS version 1.3.4 (9). Previously, we reported the assembly of two complete *Dehalobacter* genomes from these two metagenomes (3). The contigs from the subculture metagenome that mapped to either of the two *Dehalobacter* genomes were removed to obtain the contigs of microbes other than *Dehalobacter* spp. These contigs were compared to scaffolds generated from the ACT-3 metagenome. A circular scaffold comprising twenty non-*Dehalobacter* contigs was constructed. Nineteen gaps were resolved *in silico* with a previously described method (3). The last gap, a highly repetitive region, was resolved by long reads generated from additional Sanger sequencing guided by PCR. Having two metagenome data sets enabled the complete assembly: the ACT-3 metagenome provided long-distance mate-pair constraints for scaffolding despite having insufficient coverage (~14×) to close the genome, while the CF metagenome provided sufficient coverage (~40×).

The genome of *Bacteroidales* strain CF is ~2.66 Mb, with an average G+C content of 42.7%. It contains 9 rRNA genes (3 16S rRNA genes), 37 tRNA genes, and ~2,310 coding sequences. It was annotated with RAST (10) and BASys (11). The genes required for the fermentation of l-lactate, ethanol, and glucose were identified. Genes encoding various hydrogenases and an incomplete citric acid cycle were also found. No essential genes involved in the Wood-Ljungdahl pathway were identified. The closest genome sequence is a draft genome sequence of *Alistipes putredinis* DSM 17216 (GenBank accession no. ABFK00000000), whose 16S rRNA sequences share only ~86% identity with those of *Bacteroidales* strain CF. 

### Nucleotide sequence accession number.

This genome sequence has been deposited in GenBank with the accession no. CP006772.
